# Supplementary material for: End Sequence Analysis Toolkit (ESAT) expands the extractable information from single-cell RNA-seq data
Source: Genome Res. 2016 Oct;26(10):1397–410. doi: 10.1101/gr.207902.116 (PMC5052061; doi:10.1101/gr.207902.116)
Supplement: Supplemental Material [file supp_gr.207902.116_Supp_Materials.tar.gz › ESAT-esat_v0.1/doc/html/classbroad_1_1pda_1_1seq_1_1segmentation_1_1_global_splice_filter-members.html]

ESAT: Member List


ESAT


- Main Page
- Related Pages
- Packages
- Classes
- Files

- Class List
- Class Index
- Class Hierarchy
- Class Members

All Classes Namespaces Files Functions Variables Enumerator Pages

- broad
- pda
- seq
- segmentation
- GlobalSpliceFilter

broad.pda.seq.segmentation.GlobalSpliceFilter Member List

This is the complete list of members for broad.pda.seq.segmentation.GlobalSpliceFilter, including all inherited members.

|  |  |  |
| --- | --- | --- |
| GlobalSpliceFilter(AlignmentDataModel data, String save, String genomeDirectory, String chr) | broad.pda.seq.segmentation.GlobalSpliceFilter |  |
| main(String[] args) | broad.pda.seq.segmentation.GlobalSpliceFilter | static |


---

Generated on Fri Aug 15 2014 15:11:05 for ESAT by  

 1.8.7
